# Cynipid wasps systematically reprogram host metabolism and restructure cell walls in developing galls

**DOI:** 10.1093/plphys/kiae001

**Published:** 2024-01-17

**Authors:** Kasey Markel, Vlastimil Novak, Benjamin P Bowen, Yang Tian, Yi-Chun Chen, Sasilada Sirirungruang, Andy Zhou, Katherine B Louie, Trent R Northen, Aymerick Eudes, Henrik V Scheller, Patrick M Shih

**Affiliations:** Department of Plant and Microbial Biology, University of California, Berkeley, Berkeley, CA 94720, USA; Feedstocks Division, Joint BioEnergy Institute, Emeryville, CA 94608, USA; Environmental Genomics and Systems Biology Division, Lawrence Berkeley National Laboratory, Berkeley, CA 94608, USA; Environmental Genomics and Systems Biology Division, Lawrence Berkeley National Laboratory, Berkeley, CA 94608, USA; Environmental Genomics and Systems Biology Division, Lawrence Berkeley National Laboratory, Berkeley, CA 94608, USA; Joint Genome Institute, Lawrence Berkeley National Laboratory, Berkeley, CA 94720, USA; Feedstocks Division, Joint BioEnergy Institute, Emeryville, CA 94608, USA; Environmental Genomics and Systems Biology Division, Lawrence Berkeley National Laboratory, Berkeley, CA 94608, USA; Feedstocks Division, Joint BioEnergy Institute, Emeryville, CA 94608, USA; Environmental Genomics and Systems Biology Division, Lawrence Berkeley National Laboratory, Berkeley, CA 94608, USA; Department of Plant and Microbial Biology, University of California, Berkeley, Berkeley, CA 94720, USA; Feedstocks Division, Joint BioEnergy Institute, Emeryville, CA 94608, USA; Environmental Genomics and Systems Biology Division, Lawrence Berkeley National Laboratory, Berkeley, CA 94608, USA; Center for Biomolecular Structure, Function and Application, Suranaree University of Technology, Nakhon Ratchasima 30000, Thailand; Department of Plant and Microbial Biology, University of California, Berkeley, Berkeley, CA 94720, USA; Feedstocks Division, Joint BioEnergy Institute, Emeryville, CA 94608, USA; Environmental Genomics and Systems Biology Division, Lawrence Berkeley National Laboratory, Berkeley, CA 94608, USA; Environmental Genomics and Systems Biology Division, Lawrence Berkeley National Laboratory, Berkeley, CA 94608, USA; Joint Genome Institute, Lawrence Berkeley National Laboratory, Berkeley, CA 94720, USA; Environmental Genomics and Systems Biology Division, Lawrence Berkeley National Laboratory, Berkeley, CA 94608, USA; Joint Genome Institute, Lawrence Berkeley National Laboratory, Berkeley, CA 94720, USA; Feedstocks Division, Joint BioEnergy Institute, Emeryville, CA 94608, USA; Environmental Genomics and Systems Biology Division, Lawrence Berkeley National Laboratory, Berkeley, CA 94608, USA; Department of Plant and Microbial Biology, University of California, Berkeley, Berkeley, CA 94720, USA; Feedstocks Division, Joint BioEnergy Institute, Emeryville, CA 94608, USA; Environmental Genomics and Systems Biology Division, Lawrence Berkeley National Laboratory, Berkeley, CA 94608, USA; Department of Plant and Microbial Biology, University of California, Berkeley, Berkeley, CA 94720, USA; Feedstocks Division, Joint BioEnergy Institute, Emeryville, CA 94608, USA; Environmental Genomics and Systems Biology Division, Lawrence Berkeley National Laboratory, Berkeley, CA 94608, USA; Joint Genome Institute, Lawrence Berkeley National Laboratory, Berkeley, CA 94720, USA; Innovative Genomics Institute, University of California, Berkeley, CA 94720, USA

## Abstract

Many insects have evolved the ability to manipulate plant growth to generate extraordinary structures called galls, in which insect larva can develop while being sheltered and feeding on the plant. In particular, cynipid (Hymenoptera: Cynipidae) wasps have evolved to form morphologically complex galls and generate an astonishing array of gall shapes, colors, and sizes. However, the biochemical basis underlying these remarkable cellular and developmental transformations remains poorly understood. A key determinant in plant cellular development is cell wall deposition that dictates the physical form and physiological function of newly developing cells, tissues, and organs. However, it is unclear to what degree cell walls are restructured to initiate and support the formation of new gall tissue. Here, we characterize the molecular alterations underlying gall development using a combination of metabolomic, histological, and biochemical techniques to elucidate how valley oak (*Quercus lobata*) leaf cells are reprogrammed to form galls. Strikingly, gall development involves an exceptionally coordinated spatial deposition of lignin and xylan to form de novo gall vasculature. Our results highlight how cynipid wasps can radically change the metabolite profile and restructure the cell wall to enable the formation of galls, providing insights into the mechanism of gall induction and the extent to which plants can be entirely reprogrammed to form unique structures and organs.

## Teaser

Cynipid wasps radically change the metabolite profile and restructure the cell wall to enable the formation of galls.

## Introduction

Diverse organisms from fungi and bacteria to plants and insects have independently evolved the ability to manipulate the growth of plants to their advantage, forming abnormal structures referred to as galls. Galls induced by bacteria and fungi are generally morphologically simple and often referred to simply as “tumors,” whereas galls induced by insects are sometimes intricately and precisely structured (Gatjens-Boniche 2019) and have fascinated naturalists since the time of ancient Greece (Theophrastus 1976). While the exact mechanisms of gall induction remain largely unknown, chemical signals from the insect causing plant growth reprogramming has been the primary working hypothesis since the time of Charles Darwin, who presented “the poison secreted by the gall-fly produces monstrous growths on the wild rose or oak-tree” as one of the final arguments suggesting all plants (and in fact all life) share common ancestry (Darwin 1859). Interestingly, some gall inducers create galls on several species of host plants, and in these cases, the gall morphology is remarkably similar (Russo 2021). This demonstrates that the gall is an extended phenotype of the gall inducer (Dawkins 1978; Stone et al. 2003), which exerts greater control over gall morphology than the plant host. The genes underlying this extended phenotype in insect gall inducers remain almost entirely unknown, but the phenotype itself is striking: precise control over host growth, metabolism, and structure.

Deciphering the mechanisms of gall induction has been a longstanding goal of gall research (McCalla et al. 1962; Gatjens-Boniche 2019). One major theory is that gall inducers synthesize plant hormones or hormone analogs, the local concentration gradients of which play a key role in gall development. Synthesis of plant hormones—principally auxin and cytokinin—is known to be a key component in the generation of the simple galls induced by *Agrobacterium* (Nester 2015). Nonetheless, hormones likely play some role in insect gall induction, a hypothesis supported by the detection of high concentrations of plant hormone analogs in gall tissue (Yokota et al. 1982), though studies of other gall types have found galls to be auxin-depleted compared with normal tissue (Yamaguchi et al. 2012). Even more conspicuous evidence comes from the ability of some gall-inducing insects to synthesize plant hormones such as auxin and most likely cytokinin (Tooker and De Moraes 2011; Suzuki et al. 2014). Taken together, the balance of evidence suggests that phytohormone synthesis plays a role in the induction of at least some galls, but the exact mechanism is unclear and there are almost certainly other unknown elements to the induction of galls. Insect-produced effector proteins are a recently discovered candidate for those additional elements (Korgaonkar et al. 2021). In particular, simple concentration gradients of hormones are insufficient to explain the morphological diversity and complexity of insect-induced galls; thus there is a need to discover, study, and expand our understanding of the many nonphytohormone compounds that may contribute to the development and morphology of complex galls.

Because cell walls physically surround and constrain plant cells, any new growth such as the development of a gall requires breakdown, remodeling, and/or new deposition of cell wall material. As such, cell wall remodeling is a key to organogenesis, such as the generation of new leaves (Traas 2018). Despite the central role cell walls play in determining the structure and function of plant tissues, little is known about how plant cell walls are modified during the development of insect galls. Previous qualitative studies have shown changes in lignin (Tanaka et al. 2013), tannins (Jankiewicz et al. 2021), and several polysaccharides (Formiga et al. 2013; Martini et al. 2019); however, there is a need for a more global understanding of all the metabolic changes underlying the transformation of the plant cell metabolites and cell walls during the formation of galls. Similarly, a more detailed spatial understanding of the molecular alteration in plant cell walls associated with gall induction may reveal unique insights into the relatively unexplored interplay between host cell reprogramming and cell wall remodeling.

Among the most morphologically complex and charismatic galls are those induced by cynipid wasps on oak (*Quercus* spp.) trees (Stone and Cook 1998). Over 1,300 species of cynipid wasps have been described (Ronquist and Liljeblad 2001), and many species alternate between a sexual and parthenogenic generation, each of which produces a distinct gall type (Harper et al. 2004). The diversity and morphological complexity of cynipid galls make them an excellent system to study the morphological, metabolic, and cell wall changes associated with gall induction. Recent molecular biology research on cynipid galls has been largely limited to transcriptomics studies (Hearn et al. 2019; Martinson et al. 2022). These analyses have shed some light on the question of how cynipid wasps hijack the gene expression machinery of plants, but the metabolic consequences of these changes in gene expression remain largely unexplored. However, because insect gall induction is believed to be dependent on the generation of gradients of signaling molecules such as phytohormones and requires changes to cell wall structure and composition without obvious mRNA proxies, transcriptomics alone cannot tell the whole story. To provide a more comprehensive understanding of oak gall development, we perform a detailed analysis of the biochemical changes associated with gall induction and the concurrent alterations to cell wall structure and composition.

## Results

### Morphological characterization of 2 distinct gall types

**Figure 1.**
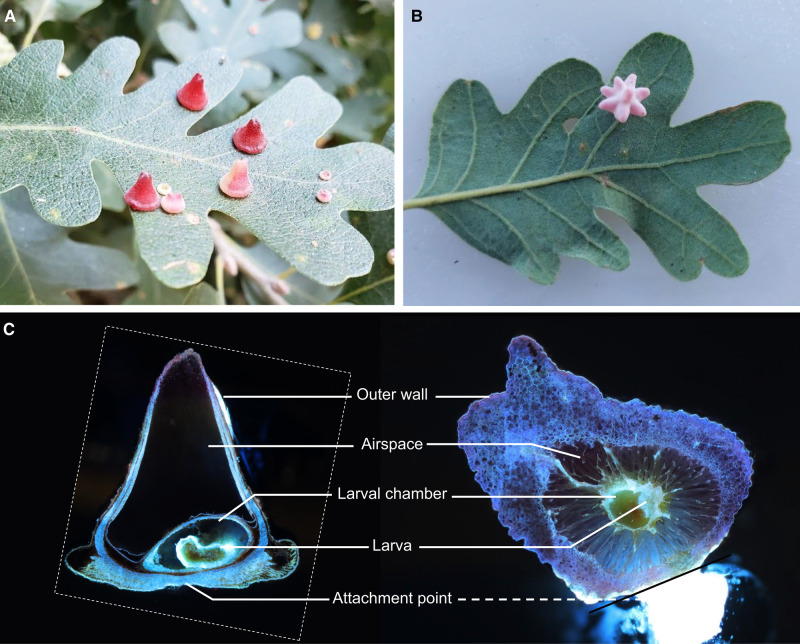
Comparison of anatomical features shared across morphologically disparate galls. **A)** Cone galls induced by *And. kingi* on the valley oak *Q. lobata*. **B)** Urchin gall induced by *A. douglasii*, also on *Q. lobata*. **C)** Longitudinal sections of cone (left) and urchin (right) galls, imaged with LAT. The dashed line to the urchin gall attachment point indicates attachment point is out of the plane and location is approximate. The dashed outline indicates the outline of the cone gall image. The bright object below and to the right of the black line on urchin gall is not part of the biological sample, and it is the support structure to which the sample was attached for imaging purposes.

We collected cone galls induced by *Andricus kingi* and urchin galls induced by *Antron douglasii* from the leaves of the valley oak *Quercus lobata* in and near the UC Davis arboretum (trees sampled shown in Supplementary Fig. S1; sampling dates and other galls identified shown in Supplementary Data Set 1). Cone galls (Fig. 1A) are cone shaped, usually red but often white along one side, and ∼5 mm across at maturity. Urchin galls (Fig. 1B) are rarer and larger, light purple in color, and urchin-shaped with 5 to 15 spikes. Both are attached to the leaf by a thin (<200 *µ*m) section of tissue that projects orthogonal to the plane of the leaf blade and defines the axis of rotational symmetry for cone galls and approximate symmetry for urchin galls.

Galls induced by cynipid wasps are complex 3D structures; however, the vast majority of studies have been constrained to 2D sections, which has been insufficient to comprehensively characterize the relationship between plant and insect tissue. To fill this gap, we used laser ablation tomography (LAT) to generate high-resolution 3D reconstructions of galls consisting of thousands of 2D slices (Fig. 1C; Supplementary Fig. S2). Three-dimensional models reveal the internal structure of cone (Videos 1 and 2) and urchin (Video 3) galls. The *And. kingi* larva within the cone galls is highly autofluorescent, facilitating easy discrimination between larval and plant tissue. Surprisingly, we found the larva in different orientations in the 2 cone galls imaged, with the long axis parallel to the longitudinal axis of the cone gall in one case and perpendicular in the other. This variation in the orientation of the larval chamber in conjunction with the tight conservation of the overall gall structure suggests something other than the larva provides the “orientation lodestar” for gall development, most likely the attachment point to the leaf.

While the morphology of both types of gall is very different, at maturity, they both consist of a relatively thick outer wall of plant cells, an airspace, and an inner layer of plant cells surrounding a fluid-filled cavity, which houses the developing larva. The urchin gall larval chamber is suspended by thin strands of plant tissue in the center of the airspace, providing thermal insulation. The thick exterior wall and airspace have been demonstrated to be important for the protection of the larva from the elements (Miller et al. 2009) and hypothesized to be important for protection from predators and parasitoids (Stone and Cook 1998). An evolutionary arms race between gall inducers and these natural enemies is likely a contributing source of the tremendous variation in cynipid gall morphology.

The 3D models show that plant epidermal cells surrounding the larval chamber are patterned in a smooth ovate structure. While the galls imaged with LAT were relatively mature, the insect larva remained fairly undeveloped and likely incapable of chewing through the plant cells surrounding the chamber. However, they were within an order of magnitude of the size of the adult wasps, which means they had almost certainly grown quite substantially to reach their current size. These facts together support the hypothesis that up to and including this gall developmental stage, insect larvae are absorbing nutrients through the fluid within the larval chamber, much like other animals feed on energy reserves within an egg or plant seedlings feed on endosperm, and in contrast to the mechanical chewing found in galling thrips (Crespi et al. 1997) and during the final stages of cynipid development (Shorthouse and Rohfritsch 1992). The fluid of the larval chamber is most likely to be translocated photosynthate and nutrients, highlighting the importance of vasculature to support proper gall development.

### Metabolomic profiles of different gall types are distinct and unique

We examined the metabolomic profiles of the 2 gall types, looking for common patterns that may suggest the homologously shared mechanism of gall induction as well as differences that may explain the differences in gall morphology. Previous research has focused either on a small number of preselected metabolites (Hartley 1998; Allison and Schultz 2005; Kot et al. 2018a) or on the transcriptional profile of galls (Hearn et al. 2019; Martinson et al. 2022). We utilized untargeted metabolomics to quantify thousands of mass features in galls and ungalled leaf tissues. The most recent common ancestor of the 2 species of gall wasp studied most likely also induced galls (Ronquist et al. 2015), and therefore, shared changes in the metabolomic profile of the 2 galls may suggest key elements of the basic mechanism of gall induction, whereas differences between the 2 galls may be either a cause or result of more idiosyncratic elements of gall structure or random changes due to drift.

**Figure 2.**
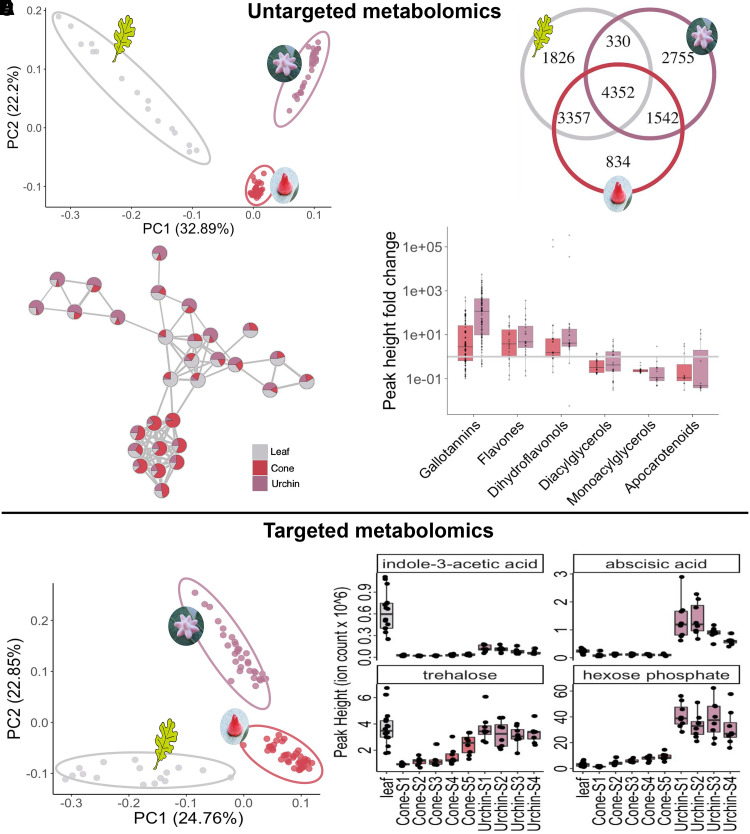
Galls are metabolically distinct from each other and leaf tissue. **A)** Principal component analysis of all 8,690 mass features recorded in positive mode in untargeted metabolomics, pooling all growth stages for each gall type. **B)** Venn diagram of the mass features present in each sample type in untargeted metabolomics. **C)** Molecular networking, showing one subnetwork without node labels. Each node is a mass feature, and each edge indicates a cosine score of fragmentation pattern of at least 0.7, with edge thickness corresponding to cosine score with maximum thickness at cosine = 1. Nodes are pie charts indicating relative peak heights between the 3 sample types. Full interactive networks can be viewed online at NDExbio, and additional networks can be viewed in Supplementary Fig. S4. **D)** Natural products classes of putative identifications of mass features in untargeted metabolomics. Each point shows the average fold-change of a particular putatively identified mass feature within the class; boxplots extend from 25th to 75th percentile of fold-change within each class, with a line at the median; whiskers extending 1.5× the interquartile range; and raw data plotted as points. **E)** Principal component analysis of all 209 metabolites positively identified with mass charge ratio, secondary fragmentation pattern, and RT confirmed against a library for the same instrument in positive and negative modes, removing whichever was lower to generate a nonredundant dataset. **F)** Metabolite data for leaf and several growth stages of each type of gall for 2 hormones and 2 sugars. MS–MS mirror plots with more precise identification information of abscisic acid, trehalose, and hexose phosphate are available in Supplementary Figs. S6 to S8, respectively. Indole-3-acetic acid peak height was too low to trigger MS–MS; identification was based on RT, m/z ratio, and other mass feature characteristics shown in Supplementary Data Set 4.

Metabolic changes associated with the initial induction of galls are expected to be especially pronounced during the early stages of gall development. Therefore, cone and urchin galls were subdivided into 5 and 4 developmental stages, respectively, using mass as a proxy for the developmental stage (Materials and methods; Supplementary Fig. S1). The resulting dataset enables the metabolomic analysis of cynipid gall development incorporating a developmental time-series design. We obtained 8,690 mass features; the full datasets for positive and negative MS modes are available as Supplementary Data Sets 2 and 3, respectively; the heat map of mass feature peak height is available in Supplementary Fig. S3. Principal component analysis demonstrated that mass feature composition was distinct for leaf, urchin gall, and cone gall samples (Fig. 2A). The majority (63%) of these mass features were shared between at least 2 sample types, and 29% were shared among all 3, leaf and both galls (Fig. 2B).

We performed network analysis using Global Natural Product Social Molecular Networking (GNPS), which revealed that mass features overrepresented in particular sample types often clustered, demonstrating similar classes of compounds were enriched in specific galls (Fig. 2C; Supplementary Fig. S3). Several interactive networks are available online at NDExbio—further described in Materials and methods and additional networks in Supplementary Fig. S4. We used the *m/z* ratio and networking to generate putative identifications for each mass feature and used a Natural Product Classifier (NPClassifier) to categorize them (Kim et al. 2021), revealing increases in several expected compound classes such as gallotannins (whose name derives from “gall”) in gall tissue compared with leaf. We also observed an increase in 2 flavonoid categories and a decrease in 2 acylglycerol categories as well as in apocarotenoids (Fig. 2D; Supplementary Fig. S5). Our finding of increased flavonoid accumulation corroborates a recent report of the upregulation of flavonoid biosynthetic genes in cynipid oak galls (Martinson et al. 2022), which also may be the underlying basis of the pigmentation of the galls themselves. The decrease in acylglycerols is consistent with the same study observing that 2 of the top 50 upregulated genes were annotated as “hydrolysis of fatty acids.” It has been proposed that fatty acids are converted into sugars to feed the growing larva (Martinson et al. 2022). Finally, cynipid galls have previously been shown to contain lower concentrations of chlorophyll and carotenoids (Kot et al. 2018b, 2020), suggesting reduced photosynthesis as an explanation for the reduction in apocarotenoids observed here.

To provide a more detailed and quantitative understanding of metabolite changes, we next performed targeted metabolomics based on a library of standards with known retention time (RT) and fragmentation data to identify specific metabolites that broadly cover a wide sampling of primary metabolism and many core plant metabolites. Targeted metabolomic analyses combined the positive-mode and negative-mode datasets by choosing whichever had higher peak height [following methodology from (Calderón-Santiago et al. 2016)], resulting in a nonredundant dataset of 209 metabolites with a confidence score of at least “Level 1,” meaning at least 2 independent and orthogonal data are used to confirm metabolite identity (Sumner et al. 2007). Identification evidence including MS1, MS2, and chromatographic peak comparisons are available as Supplementary Data Sets 4 and 5 for positive and negative modes, respectively. The principal component analysis of this stringently curated dataset revealed a distinct separation of sample types (Fig. 2E), and the full dataset is available as Supplementary Data Set 6. Additional principal component analyses of the growth stages of each type of gall reinforce a clear distinction between gall and leaf metabolites and show partial clustering by gall growth stage (Supplementary Fig. S3).

Of these 206 identified metabolites from targeted metabolomics, 39 had peak heights averaged across all growth stages of urchin galls >4 times higher than the leaf average, and 22 had peak heights in cone galls >4 times higher than the leaf average. Of these highly gall-abundant metabolites, 11 were enriched in both gall types, much more than would be predicted if peak heights were independent in both gall types (*P* = 0.0005, hypergeometric test). Peak height data for the 54 metabolites >4× higher peak height in galls compared with leaf tissue is available in Supplementary Data Set 7. These metabolites are candidates for either causes or conserved metabolic effects of the gall induction process and may be useful leads for future efforts to determine the mechanism of gall induction. We used NPClassifier to classify all 209 metabolites by pathway and evaluated whether any pathways were overrepresented among the metabolites enriched in galls. For both gall types, there were fewer fatty acids than chance (*P* = 0.052 for cone galls and *P* = 0.024 for urchin galls, hypergeometric test), supporting the results from the untargeted metabolomics. All NPClassifier terpenoid categories (Bisaboline sesquiterpenoids, Labdane diterpenoids, Farnesane sesquiterpenoids, and 6 other terpenoid classes; Supplementary Fig. S5) were reduced in galls, which we speculate may reflect the downregulation of plant defenses by the wasp larvae.

### Conserved metabolite changes across different galls reveal drastic changes in plant hormone and sugar concentrations

We next examined the concentration of plant hormones detected in the metabolomic analyses, which have been hypothesized to play important roles in the gall induction process. Structurally complex galls can be thought of as a novel organ functioning for the benefit of the gall inducer, and hormone concentration gradients are known to be central to the growth of organs such as leaves, flowers, and fruits. Interestingly, the transcriptomic profile of galls induced by phylloxera on grape leaves shares many similarities with the transcriptome of fruits (Schultz et al. 2019).

We found major differences in the concentration of auxin (indole-3-acetic acid) and abscisic acid between galls and ungalled leaf tissues of comparable age found nearby (Fig. 2F). Existing literature shows that auxin and cytokinin are sometimes increased and sometimes decreased in gall tissue compared with normal plant tissue, suggesting there may be multiple separate mechanisms of plant growth manipulation used by different groups of gall inducers (reviewed in Tooker and Helms 2014). This is not surprising given that the gall-inducing habit has evolved independently many times in separate lineages (Raman et al. 2006 ). In both cone and urchin galls, we see a massive decrease in the concentration of auxin (Fig. 2F). This is somewhat surprising given the relatively low baseline levels of auxin in the middle of a leaf lamina (Kojima et al. 2009) and even more surprising in light of the fact that RNAseq of a closely related cynipid-induced oak gall showed the upregulation of auxin-response genes (Hearn et al. 2019). While it is possible that these discordant results reflect different ground truths in these closely related cynipid galls, it is also possible that the upregulation of auxin biosynthetic genes does not result in increased auxin accumulation, highlighting a potential pitfall of interpretations of small molecule concentration solely made by transcript levels without direct biochemical measurement.

Abscisic acid concentration is increased in urchin galls, but not cone galls (Fig. 2F). Abscisic acid is often associated with stress and has been shown to increase in response to attempted gall induction on resistant plants while remaining constant between gall and normal tissue in susceptible plants (Tokuda et al. 2013). In another gall system, abscisic acid was reported to be decreased in gall tissue compared with normal plant tissue (Zhu et al. 2011). In light of these diverging results among very phylogenetically distant gall systems, it is interesting to see different behavior in abscisic acid response even among 2 closely related galls on the same plant host. It is also worth noting that the only mass features identified as apocarotenoids increased in gall tissue in Fig. 2D were putatively identified as abscisic acid. Since this was an independent MS run, that both strengthen the results from this targeted analysis, and their removal from the apocarotenoid class (of which abscisic acid is clearly a noncentral example) strengthens the finding that apocarotenoids are depleted in gall tissue.

We also observed a striking pattern in the concentration of trehalose, a disaccharide known to play important signaling and regulatory roles. Trehalose mediates plant immunity: trehalose synthesis mutants are more vulnerable to aphids (Singh et al. 2011), and exogenous application of trehalose induces resistance against pathogens (Tayeh et al. 2014). The massive reduction of trehalose concentration in cone galls may suggest the wasps are silencing this defense response. Trehalose also plays important roles in insects; it is a major circulating carbohydrate in the hemolymph (2003), as well as a regulator of long-term hibernation-like states (Li et al. 2020). Therefore, further research is necessary to fully understand the implications of the trehalose reduction in gall tissue.

We next examined hexose phosphates, central metabolic intermediates, which are a primary output of photosynthesis and primary input into the cell wall assembly. Hexose phosphates are substantially enriched in all surveyed developmental stages of urchin gall tissue compared with leaf but remain constant at leaf-like levels in cone galls (Fig. 2F). On average, hexose phosphate levels in urchin galls are over 10 times higher than the leaf baseline. In general, the majority of hexose phosphates are destined for the generation of starch or cell wall polysaccharides, suggesting the rerouting of metabolism to support gall development and larval feeding.

### Gall cell layers are chemically distinct and highly lignified suggestive of de novo vascularization

**Figure 3.**
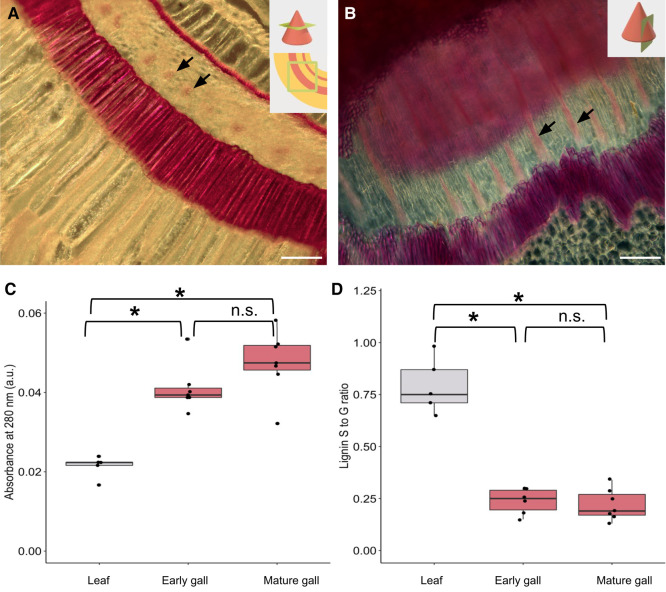
Lignin deposition in cone galls is spatially coordinated in a gall-specific pattern. **A)** Transverse section of cone gall stained with the Wiesner stain, showing 2 heavily lignified cell layers and one cell layer containing bundles of 4 to 9 highly lignified cells (arrows). Scale bar = 100 *µ*m. **B)** Darkfield image of a tangential longitudinal section of gall stained with the Wiesner stain, which stains heavily lignified tissue with pink. The same 2 heavily lignified cell layers are visible, as well as the small moderately lignified bundles (arrows), now in a longitudinal section. Scale bar = 100 *µ*m. **C)** Lignin concentration in leaf tissue, early-development cone galls, and mature cone galls, as determined by TGA assay. Boxplot center line indicates median, box limits indicate 25th and 75th percentiles, whiskers extend 1.5× the interquartile range, and points are raw data. Asterisks indicate *P* < 0.05 by Kruskal–Wallis test with Benjamini–Hochberg correction for multiple comparison; n.s. indicates *P* > 0.05. **D)** Lignin subunit S-to-G (syringyl to guaiacyl) ratio as determined by pyro-GC MS. Asterisks indicate *P* < 0.05 by Kruskal–Wallis test with Benjamini–Hochberg correction for multiple comparison; n.s. indicates *P* > 0.05.

Though the 3D models generated by LAT offer unique structural insights, they lack chemical information. Metabolomic analysis offers chemical information, but without spatial data. To address the intersection of these interests, we turned to histochemical staining. Histochemical staining is a standard approach to identifying plant tissue types, yet there are no published micrographs of either of the galls studied here. Therefore, we next used a series of classic plant histology stains on cone galls (chosen for microscopy as they were more abundant) to examine the chemical composition and distribution to better understand the chemical changes associated with gall development. Safranin O, Congo red, Mäule stain, cellulose azure, orange G, FastGreen FCF, and aniline blue failed to show any interesting spatial patterns within the gall material (Supplementary Fig. S9). Toluidine blue was useful for generating contrast to determine cell wall morphology and differentiate cell layers (Supplementary Fig. S10). Wiesner reagent (phloroglucinol + HCl) revealed the most striking spatial pattern, demonstrating the tight spatial regulation of lignin deposition in gall tissue (Fig. 3, A and B). Two sclerenchyma cell layers are strongly stained (Supplementary Fig. S10A), and the central sponge layer between them contains bundles of 4 to 9 cells in cross-section with moderate lignification, which is suggestive of vasculature.

Wiesner staining revealed large amounts of lignin, but histological studies cannot provide an accurate quantification of these chemical changes. To fill this gap, we used the thioglycolic acid (TGA) assay to quantify lignin in leaf and gall tissues, comparing leaf tissue against young or mature cone galls, as shown in Fig. 3C. Cone galls are substantially more lignified than leaf tissue (*P* = 0.0025, Kruskal–Wallis test with Benjamini–Hochberg correction for multiple comparisons), but the 2 developmental stages are statistically indistinguishable from each other. In light of this substantial increase in lignin levels, we asked whether the lignin monomeric composition was altered as well using pyrolysis gas chromatography coupled to MS (pyro-GC MS). Lignin polymers are composed of 3 subunits, namely syringyl (S), guaiacyl (G), and p-hydroxyphenyl (H), which polymerize with a complex branched structure that is highly resistant to degradation (Weng and Chapple 2010; Li et al. 2016). Lignin associated with fiber cells tends to contain a higher fraction of S subunits, whereas vascular elements contain more G subunits (Nakashima et al. 2008). The S-to-G ratio was substantially lower in both stages of gall tissue (Fig. 3D, *P* = 0.011 and 0.0076 for early and mature galls respectively, Kruskal–Wallis test with Benjamini–Hochberg correction for multiple comparisons; full data for all lignin-derived fragments are available in Supplementary Data Set 8) compared with leaf tissue, which also supports the generation of vasculature in the galls, though we cannot rule out changes in other tissue types such as fiber cells contributing to the monomeric composition change.

Our findings are in contrast to a detailed analysis of another cynipid-induced gall, where de novo production of vasculature was specifically ruled out (Brooks and Shorthouse 1998), suggesting neovascularization only occurs in some types of cynipid galls. Obtaining access to the plant vascular system has long been recognized as important for the growth and success of galling insects (Wool et al. 1999), but previous studies have shown modifications of existing vasculature rather than de novo vascularization. In contrast, the histological evidence here demonstrates gall generation involves the coordinated, spatially organized generation of de novo vasculature.

### Cell wall remodeling is associated with gall formation

**Figure 4.**
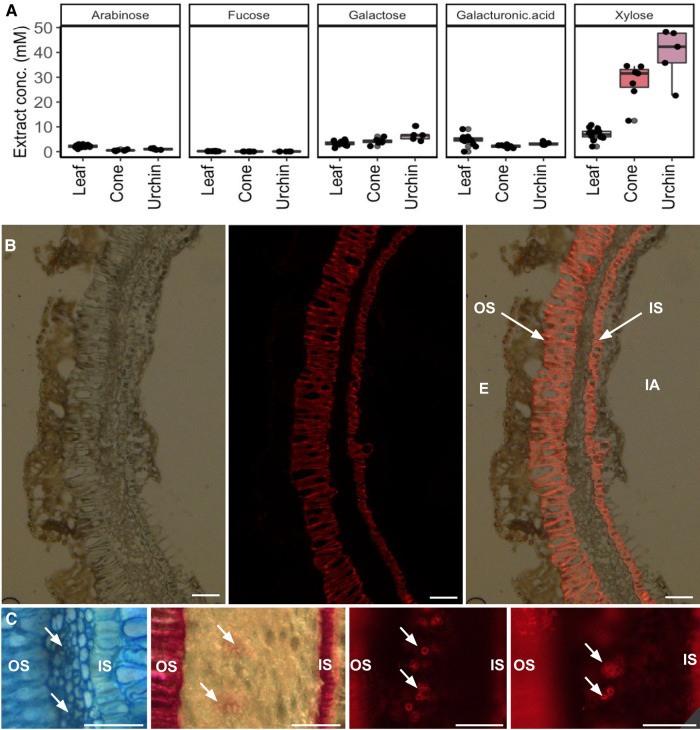
The composition of gall cell walls is altered to be highly enriched in xylan. **A)** Concentration of 5 sugars in cell wall residue hydrolysate. Glucose potentially derived from cell wall polymers cannot be accurately measured due to starch contamination. Boxplot center line indicates median, box limits indicate 25th and 75th percentiles, whiskers extend 1.5× the interquartile range, and points are raw data. **B)** LM10 immunofluorescence staining signal for xylan. Left: differential interference contrast transmitted light. Center: Alexa-fluor 647 secondary antibody conjugated to LM10 primary antibody. Right: overlay. OS, outer sclerenchyma; IS, inner sclerenchyma; E, exterior; IA, interior airspace. Scale bar = 50 *µ*m. **C)** Views of vascular bundles in sponge layer, from left to right: toluidine blue O, Wiesner stain, LM10, LM10. In each case, the outer sclerenchyma cells are shown on the left, the sponge layer containing vascular bundles (arrows) in the middle, and the inner sclerenchyma on the right (mostly cropped out in LM10 images due to focus and saturation issues). All scale bars = 50 *µ*m; uncropped source images are available in Supplementary Fig. S11.

The cell wall plays an integral role in defining the form and function of plant cells. Although we had already observed changes in lignin composition and deposition, the majority of the cell wall is composed of polysaccharides. Changes in polysaccharide content can drastically alter the biochemical, physical, and ultimately physiological role of plant cells and tissues. A large portion of plant sugars are ultimately sequestered in the cell wall as polysaccharides, which in conjunction with lignin comprise the primary physical support structure of plant organs. Since metabolomics revealed differences in hexose phosphate concentrations, we reasoned that this could lead to changes in the monosaccharide composition of the cell walls. Indeed, cell wall composition varied wildly between galls and the leaf tissue from which they arise, as shown in Fig. 4A. Notably, xylose residues were extremely abundant in gall tissue, to the extent that all other monosaccharide signals are largely suppressed, and surprisingly, xylose accounts for over 75% of all hydrolyzed cell wall monosaccharides in cone gall samples. It should be noted that the cell wall polysaccharide hydrolysis method employed leaves cellulose intact and measures the monosaccharide composition of all noncellulosic cell wall polysaccharides.

The extraordinarily high levels of xylose suggest enrichment of a polymer composed largely of xylose in gall tissue. One natural candidate is xylan, which is named after and usually enriched in xylem tissue and other vasculature fibers (Salvador et al. 2000; Kim and Daniel 2012). The antibody LM10 selectively binds to and is used to detect xylan. We performed immunofluorescence microscopy with LM10 raised in mice as primary antibodies and antimouse IgG conjugated to Alexa-fluor 647 as a secondary antibody as shown in Fig. 4B. This revealed high concentrations of xylan in the same 2 sclerenchyma cell layers, which are highly lignified (Fig. 3, A and B). The colocalization of xylan and lignin deposition is suggestive of a mechanical defense role for these 2 cell layers. Furthermore, at higher magnification and exposure, there were bundles of cells present in the sponge layer between these 2, colocalizing with the lignified bundles revealed by the Wiesner stain (arrows in Fig. 3). These bundles as viewed with toluidine blue O stain, Wiesner stain, and 2 views of LM10 immunostain are shown in orientation-matched views in Fig. 4C (uncropped source images available in Supplementary Fig. S11). The colocalization of lignin and xylan in this particular bundled spatial pattern strongly suggests these are the vascular bundles of the gall, and their less-consistent organization compared with normal vascular bundles likely reflects imperfect control of plant developmental morphology on the part of the gall-inducing wasps, as noted previously regarding the alteration of existing vasculature in galls (Taft and Bissing 1988). The cell-layer–specific alteration of lignin and polysaccharide composition—the 2 primary constituents of plant cell walls—indicates that galling insects exert a large degree of control over plant growth and metabolism in the development of galls.

## Discussion

We leveraged metabolomics, 3D light microscopy, lignin composition analysis, histology, and immunomicroscopy to study the biology of galls. In doing so, we revealed many similarities and several key differences in the metabolic and morphological changes associated with the gall induction process between 2 types of gall-inducing wasps. We observe dramatic alteration in metabolite composition in 2 gall types produced from the same tissue of the same host. While many of the changes to the metabolome are consistent across both gall types, some such as abscisic acid and hexose phosphates are strikingly different. The metabolites with consistent increases in concentration (Supplementary Data Set 7) are candidates for the shared induction mechanism of galls, whereas those with different concentration changes in the 2 gall types may be responsible for the specific gall morphology. We have further demonstrated that the cell wall lignin and polysaccharide composition of galls differs substantially from the normal plant tissue from which they arise.

We present some evidence that aligns with the previous literature on plant hormone dynamics in galls and some that contradicts previous studies. A recent gall tissue–specific RNAseq study found the upregulation of auxin biosynthetic genes only in the larval chamber tissue, which comprises a relatively small portion of the total gall biomass, with low expression of auxin-responsive genes throughout the remainder of the galls (Martinson et al. 2022). This finding may help reconcile the seemingly contradictory results: while auxin is involved somehow in the gall induction process, if only the gall larval chamber contains high concentrations of auxin, then depending on the mass ratio of the larval chamber compared with the exterior of the gall we would expect to see some reports of higher auxin concentration and some reports of lower concentration within galls, which is indeed what has been reported (Tooker and Helms 2014). Obtaining access to the plant vascular system has long been recognized as important for the growth and success of galling insects (Wool et al. 1999), but previous studies have shown modifications of existing vasculature rather than de novo vascularization. In contrast, the histological evidence here demonstrates that gall generation involves the coordinated, spatially organized generation of de novo vasculature.

We also present several lines of evidence for de novo vascularization in cone galls, a surprising finding given the leaf tissue from which the galls derive is terminally differentiated. It has long been known that gall-inducing insects modify and enlarge existing vasculature to deliver nutrients to the gall (Shorthouse and Rohfritsch 1992; Brooks and Shorthouse 1998; Wool et al. 1999). Galls induced by *Agrobacterium* were long thought to lack vasculature (Tzfira and Citovsky 2008) but were eventually shown to contain a vascular system organized somewhat differently than that found in normal plant tissue (Aloni et al. 1995; Ullrich and Aloni 2000). Leafy galls induced by *Rhodococcus fascians* have also recently been shown to induce neovascularization (Dolzblasz et al. 2018). Our finding of de novo vascularization in insect-induced galls suggests a similar slow discovery process may be at play for insect-induced galls as occurred in the history of bacteria-induced galls.

This detailed analysis of the morphological, metabolic, and structural changes found in cynipid galls invites comparison with better-understood galls such as the crown gall induced by *Agrobacterium*. The key principle of the crown gall induction by *Agrobacterium* is the transfer and expression of a relatively short stretch of “T-DNA” that comprises part of the tumor-inducing plasmid (Nester 2015). This stretch of DNA encodes enzymes in the biosynthetic pathway for auxin and cytokinin (Akiyoshi et al. 1984), which results in altered phytohormone levels and ratios in crown gall (Akiyoshi et al. 1983). The mechanism of gall induction in root-knot nematodes is less well understood, but it is notable that the gall-inducing nematodes have been shown to synthesize auxin (De Meutter et al. 2005), which suggests that the synthesis of plant hormones is a common strategy to manipulate plant tissue into expanding. Cynipid galls are much more morphologically complex than either of these better-characterized systems, and there is much more diversity in gall size, shape, location, and color. This diversity suggests that the mechanics of gall induction vary between different cynipid wasps, which is supported by our data demonstrating different changes to phytohormones. Nonetheless, the phylogenetic distribution of the galling habit within cynipid wasps suggests it is ancestral, and therefore at least some of the core mechanics are likely to be conserved (Ronquist et al. 2015).

The complex and colorful structures of galls have captured the imagination of naturalists for millennia and demonstrate a mastery of interkingdom manipulation that remains unparalleled by current plant molecular biologists. Many practices used to modify and manipulate plants are still reliant on the same techniques adapted from natural plant engineers (i.e. *Agrobacteria*) several decades ago, becoming the foundation of plant genetic transformations. Thus, looking for more examples in the nature of nonmodel, nontraditional systems to expand our perspective on the degree to which plants can be reprogrammed may inspire novel approaches to engineering plants in general. Elucidating the molecular basis of the induction of complex galls may provide the blueprint for redefining the landscape to redesigning entirely new cellular, morphological, and physiological architectures in plants.

## Materials and methods

### Gall collection

We monitored an arboretum collection of ∼100 species of oak (*Quercus* spp.) trees for galls from spring to autumn. Dozens of gall types were found, of which 2 types of galls were selected for further analysis on the basis of their morphological complexity and abundance: the cone gall induced by *And. kingi* and the urchin gall induced by *A. douglasii*, both on the valley oak *Q. lobata*. Both of these galls were found on the abaxial and adaxial surfaces of leaves between June and August of 2019 to 2022, with the cone galls being more abundant and appearing somewhat earlier. Both were markedly concentrated in particular trees; one valley oak would often contain hundreds of galls, while none could be seen on other valley oaks only a dozen meters away (Supplementary Fig. S1). Furthermore, the cone galls in particular were found to cluster on particular branches—it was common to see one branch supporting many times more galls per leaf than an adjacent branch, a somewhat surprising finding given that the gall-inducing insects can fly. Galls were collected in the UC Davis arboretum (38°31′46.0″N 121°45′45.3″W) and Putah Creek Riparian Reserve (38°31′19.7″N 121°46′50.1″W). Over 1,000 galls of these 2 species were gathered, at times individually divided into classes on the basis of mass/growth stage (Supplementary Fig. S1, B and C), at times in mass collections for large-scale metabolite analysis. Mass divisions for cone galls were as follows: 0.5 mg < cone_S1 < 1 mg < cone_S2 < 2 mg < cone_S3 < 4 mg < cone_S4 < 7 mg < cone_S5 < 10 mg. Mass divisions for urchin galls were as follows: 0.5 mg < urchin_S1 < 2 mg < urchin_S2 < 4 mg < urchin S3 < 6 mg < urchin_S4 < 10 mg. Leaf samples gathered near each gall were in the range of 4 mg < collected_mass < 7 mg. Galls were removed from the tree and flash-frozen in liquid nitrogen as quickly as possible. The date of collection and the specific tree of origin were noted for each gall sample (Supplementary Data Set 1 and Fig. S1).

### Laser ablation tomography

Fresh gall samples were sent to LATscan (State College, PA, USA) to perform LAT. In brief, samples are attached to a piece of pasta as a sacrificial supporting structure and then mounted in the beam path of a microscope from the front and a high-power flat-beam laser from the side. Rapid alternation of microscope image captures, and laser pulses allows for the rapid acquisition of several thousand serial “slice” images through the entire sample. The resolution of slices for practical imaging purposes is ∼8 *μ*m, slices are ∼4 *μ*m apart.

### Metabolite extraction

Metabolites were extracted using a protocol adapted from Jeon et al. (2020). Galls and leaves were flash-frozen in liquid nitrogen and stored at −80 °C until processing. Samples were lyophilized and then disrupted with a steel ball in a ball mill at 30 Hz for 20 min, yielding a fine powder. The powder was weighed and then 80 *µ*L of methanol was added per mg. Samples were vortexed for 1 min and then incubated at room temperature for 20 min with continuous mixing and centrifuged at 20,000 × *g* for 5 min, and the supernatant was filtered through 0.45 *µ*m polytetrafluoroethylene (PTFE) filters.

### Mass spectrometry

In preparation for LC-MS analysis, filtered oak gall extracts were first dried in a SpeedVac (SPD111V, Thermo Scientific, Waltham, MA, USA) and then resuspended in 100% MeOH containing an internal standard mix of isotopically labeled compounds (∼15 *µ*M average of 5 to 50 *µ*M of 13C,15N Cell Free Amino Acid Mixture, #767964, Sigma; 10 *µ*g/mL 13C-trehalose, #TRE-002, Omicron; 10 *µ*g/mL 13C-mannitol, ALD-030, Omicron; 2 *µ*g/mL 13C-15N-uracil, CNLM-3917, CIL; 5.5 *µ*g/mL 15N-inosine, NLM-4264, CIL; 4 *µ*g/mL 15N-adenine, NLM-6924, CIL; 3 *µ*g/mL 15N-hypoxanthine, NLM-8500, CIL; 5 *µ*g/mL 13C-15N-cytosine, #294108, Sigma; 2.5 *µ*g/mL 13C-15N-thymine, CNLM-6945, CIL; 1 *µ*g/mL 2-amino-3-bromo-5-methylbenzoic acid, R435902, Sigma), with resuspension volume of each varied to normalize by biomass for each sample group.

Ultra-high-performance liquid chromatography (UHPLC) normal phase chromatography was performed using an Agilent 1290 LC stack, with MS and MS/MS data collected using a QExactive HF Orbitrap MS (Thermo Scientific, San Jose, CA, USA). Full MS spectra were collected from *m/z* 70 to 1,050 at 60 k resolution in both positive and negative ionization modes, with MS/MS fragmentation data acquired using stepped then averaged 10, 20, and 40 eV collision energies at 15,000 resolution. MS source settings included a sheath gas flow rate of 55 (au), auxiliary gas flow of 20 (au), spray voltage of 3 kV (for both positive and negative ionization modes), and capillary temperature of 400 °C. Normal phase chromatography was performed using a HILIC column (InfinityLab Poroshell 120 HILIC-Z, 2.1 × 150 mm, 2.7 *µ*m, Agilent, #683775-924) at a flow rate of 0.45 mL/min with a 3 *μ*L injection volume. To detect metabolites, samples were run on the column at 40 °C equilibrated with 100% buffer B (99.8% 95:5 [v/v] ACN:H_2_O and 0.2% acetic acid [v/v], w/5 mM ammonium acetate) for 1 min, diluting buffer B down to 89% with buffer A (99.8% H_2_O and 0.2% acetic acid [v/v], w/5 mM ammonium acetate, and 5 *µ*M methylene-di-phosphonic acid) over 10 min, down to 70% (v/v) over 4.75 min, down to 20% (v/v) over 0.5 min, and isocratic elution for 2.25 min, followed by column reequilibration by returning to 100% B over 0.1 min and isocratic elution for 3.9 min. The samples consisted of 8 biological replicates each, and extraction controls, with sample injection order randomized and an injection blank of 100% MeOH, were run between each sample.

Metabolite identification was based on exact mass and comparing RT and fragmentation spectra with that of standards run using the same LC-MS method. LC-MS data were analyzed using custom Python code (Yao et al. 2015), with each detected peak assigned a level of confidence, indicated by a score from 0 to 3, in the compound identification. Compounds given a positive identification had matching RT and *m/z* to that of a standard, with detected *m*/*z* ≤ 5 ppm or 0.001 Da from theoretical as well as RT ≤ 0.5 min. A compound with the highest level of positive identification (score of 3) also had matching MS/MS fragmentation spectra. An identification was invalidated when the MS/MS fragmentation spectra collected for the feature did not match that of the standard.

### Molecular networking

The LC-MS files were run via MZmine2 version 2.39 workflow to generate a list of features, which were putatively annotated using the GNPS tool (Wang et al. 2016). This pipeline produced molecular networking files for positive (13,918 features) and negative polarities (13,562). Filtering accepted features with RT > 0.6 min (post solvent front), maximum peak height >1e6, and maximum peak height fold-change between sample and extraction control >10, resulting in 8,690 and 6,305 features in negative and positive modes, respectively. The filtered features were merged into a single molecular network (14,995 nodes) created in Cytoscape software version 3.9.1 (Shannon et al. 2003; Wang et al. 2016) following a step-by-step procedure (Aron et al. 2020). The average peak height in leaf control (*n* = 16), urchin (*n* = 32), and cone (*n* = 40) galls was calculated and painted on each node as pie charts. This was followed by fold-change calculation between the average peak height of urchin or cone divided by leaf value; +1 was added to both numerator and denominator to avoid erroneous division by 0. Annotations with cosine score (MQScore) match to library compounds >0.7 were (1,886 nodes) labeled in the networks. NPClassifier was used to determine metabolite classifications of the annotations.

### Online interactive molecular networks

The molecular network of combined HILIC untargeted metabolomics without cosine thresholding: https://www.ndexbio.org/viewer/networks/02f90a6c-dafd-11ed-b4a3-005056ae23aa.

The molecular network of combined HILIC untargeted metabolomics with cosine threshold 0.7 organized by mass feature cosine score: https://www.ndexbio.org/viewer/networks/0d278c0e-dafd-11ed-b4a3-005056ae23aa.

The molecular network of combined HILIC untargeted metabolomics with cosine threshold 0.7 organized by NPClassifier class: https://www.ndexbio.org/viewer/networks/133ba370-dafd-11ed-b4a3-005056ae23aa.

### Lignin quantification

Lignin content was measured using the TGA method following Suzuki et al. (2009). One milliliter of 3 N HCl and 0.1 mL of TGA were added to 15 mg of biomass. Samples were then incubated at 80 °C for 3 h and centrifuged for 10 min at 16,100 × *g*, and the supernatant was discarded. One milliliter of sterile water was added to the pellet and vortexed for 30 s, and the sample was again centrifuged with the same conditions. One milliliter of 1 N NaOH was added to the pellet, and the sample was allowed to shake at 80 rpm at room temperature for 16 h and then centrifuged with the same conditions. One milliliter of supernatant was transferred to a new tube, and 0.2 mL of 12 N HCl was added in a fume hood. The samples were then incubated at 4 °C for 4 h and centrifuged for 10 min at 16,100 × *g*. The supernatant was discarded, and the pellet was dissolved in 1 mL of 1 N NaOH. Dilutions prepared in 1 N NaOH were used to measure absorbance (*A*_280_). Lignin concentrations were compared with the Wilcoxon rank-sum test using the Benjamini–Hochberg method for adjustment for multiple comparisons.

### Alcohol-insoluble residue preparation

Alcohol-insoluble residue preparation was adapted from Harholt et al. (2006). AIR extracts were prepared by adding ∼15 mg of flash-frozen tissue to 1 mL 100% EtOH. The tissue was then ground in a ball mill at 20 Hz for 5 min, heated at 100 °C for 30 min with periodic shaking, cooled to room temperature, and centrifuged at 21,000 × *g* for 5 min. The supernatant was discarded, and 1 mL 70% EtOH (v/v) was added and vortexed and then centrifuged at 20,000 × *g* for 1 min. These 3 steps were repeated until the supernatant was clear, and that clear supernatant was discarded. One milliliter of acetone was then added, and the samples were vortexed and centrifuged at 20,000 × *g* for 5 min; the supernatant was discarded, and the samples were dried in a speed-vac overnight. The result was a fine powder, which was stored at 4 °C.

### Lignin monomeric composition

A small amount (∼1 mg) of AIR extract was loaded into a quartz tube for Pyro-GC MS analysis using the methodology adapted from Eudes et al. (2015). Pyrolysis of biomass was performed with a Pyroprobe 5200 (CDS Analytical Inc., Oxford, PA, USA) connected with GC/MS (Thermo Electron Corporation with Trace GC Ultra and Polaris-Q MS) equipped with an Agilent HP-5MS column (30 m × 0.25 mm inner diameter and 0.25 *µ*m film thickness). The pyrolysis was carried out at 650 °C. The chromatograph was programmed from 50 °C (1 min) to 300 °C at a rate of 20 °C/min; the final temperature was held for 10 min. Helium was used as the carrier gas at a constant flow rate of 1 mL/min. The MS was operated in scan mode, and the ion source was maintained at 300 °C. The compounds were identified by comparing their mass spectra with those of the NIST library. Peak molar areas were calculated for the lignin degradation products, and the summed areas were normalized.

### Trifluoroacetic acid hydrolysis

Trifluoroacetic acid hydrolysis and high-pressure anion exchange chromatography (HPAEC) were adapted from Fang et al. (2016). A total of 5 to 10 mg of AIR was transferred to a new tube using a ±0.01 mg scale to record transferred mass. One milliliter of 2 M TFA was added to each sample in a screw-top tube and vortexed. Samples were heated to 120 °C for 1 h, vortexing for 10 s every 15 min. After cooling to room temperature, samples were centrifuged at 20,000 × *g* for 1 min, as much supernatant as possible was discarded, and the remainder was removed by speed-vac overnight. The dried pellet was dissolved in 1 mL water and shaken at 1,000 rpm at 30 °C for 1 h and then filtered through 0.45 *μ*m nitrocellulose filters. Samples were then diluted in water for HPAEC coupled with pulsed amperometric detection. As described in the text, several dilution ratios were ultimately required, ranging from 1/10 to 1/640. NaOH was used as needed to bring all samples within the range of 4 to 9 pH.

### Microscopy

Samples were either kept at 4 °C and imaged within 1 wk of collection or flash-frozen in liquid nitrogen and stored at −80 °C. Sectioning was performed with a vibratome to generate ∼50 *μ*m sections or with a cryotome to generate ∼12 *μ*m sections. While several methods of sample fixation were performed, the best results were achieved with unfixed samples embedded in 7% agarose for vibratome sectioning or “optimal cutting temperature” (Sakura Tissue-Tek OCT, part number 4583) cryotomy embedding fluid. For each stain, several concentrations and staining periods were attempted, and the most informative was selected for further work. Imaging was performed with a fluorescence microscope (Leica DM 6B) equipped with a CMOS fluorescence imaging camera (Hamamatsu ORCA-Flash4.0LT) and an RGB camera (Leica DMC4500); all images except for the immunomicroscopy are real color, with white balance adjusted as well as possible to match printed images to the image in the eyepiece. For fluorescence imaging, the Leica TXR filter cube was used in conjunction with a white-light illumination source (Leica CTR6 LED), with an excitation band of 560 ± 20 nm and emission long-pass filter with a cutoff at 610 nm.

### Data analysis

Data analysis was performed with Rstudio (Version 2022.07.0 + 548 macOS), primarily using the Tidyverse package for data manipulation and ggplot2 for visualization. Figures were assembled with Google Drawings.

## Supplementary Material

kiae001_Supplementary_Data

## Data Availability

All data produced in this project are available in the main figures, Supplementary Figures, Supplementary Data Sets, and Videos.

## Supplementary data

The following materials are available in the online version of this article.

**Supplementary Figure S1.** Gall map and photographs.

**Supplementary Figure S2.** LAT sections and 3D models.

**Supplementary Figure S3.** Gall metabolomes cluster by developmental stage.

**Supplementary Figure S4.** Gall metabolomes are distinct.

**Supplementary Figure S5.** Galls differ in chemical class concentration.

**Supplementary Figure S6.** MS–MS details for abscisic acid.

**Supplementary Figure S7.** MS–MS details for trehalose.

**Supplementary Figure S8.** MS–MS details for hexose phosphate.

**Supplementary Figure S9.** Sample of additional histological stains.

**Supplementary Figure S10.** Micrographs of cone galls and map of cell layers.

**Supplementary Figure S11.** Transverse sections of red cone galls.

**Supplementary Data Set 1.** Dates and locations of gall sampling.

**Supplementary Data Set 2.** Positive-mode untargeted MS dataset.

**Supplementary Data Set 3.** Negative-mode untargeted MS dataset.

**Supplementary Data Set 4.** Positive-mode targeted MS mass feature identification information.

**Supplementary Data Set 5.** Negative-mode targeted MS mass feature identification information.

**Supplementary Data Set 6.** Combined positive-mode and negative-mode targeted MS dataset.

**Supplementary Data Set 7.** Metabolites highly enriched in one or both types of gall.

**Supplementary Data Set 8.** Lignin subtype analysis of pyro gas chromatography MS dataset.

## Dive Curated Terms

The following phenotypic, genotypic, and functional terms are of significance to the work described in this paper:

lignin CHEBI: CHEBI:6457

TREHALOSE CHEBI: CHEBI:27082

trifluoroacetic acid CHEBI: CHEBI:45892

metabolite CHEBI: CHEBI:25212

safranin o CHEBI: CHEBI:33601
