# Supplementary material for: Cynipid wasps systematically reprogram host metabolism and restructure cell walls in developing galls
Source: Plant Physiol. 2024 Jan 17;195(1):698–712. doi: 10.1093/plphys/kiae001 (PMC11181936; doi:10.1093/plphys/kiae001)
Supplement: kiae001_Supplementary_Data [file kiae001_Supplementary_Data.zip › PP2023RA01372R1_Supplemental_Figures.pdf]

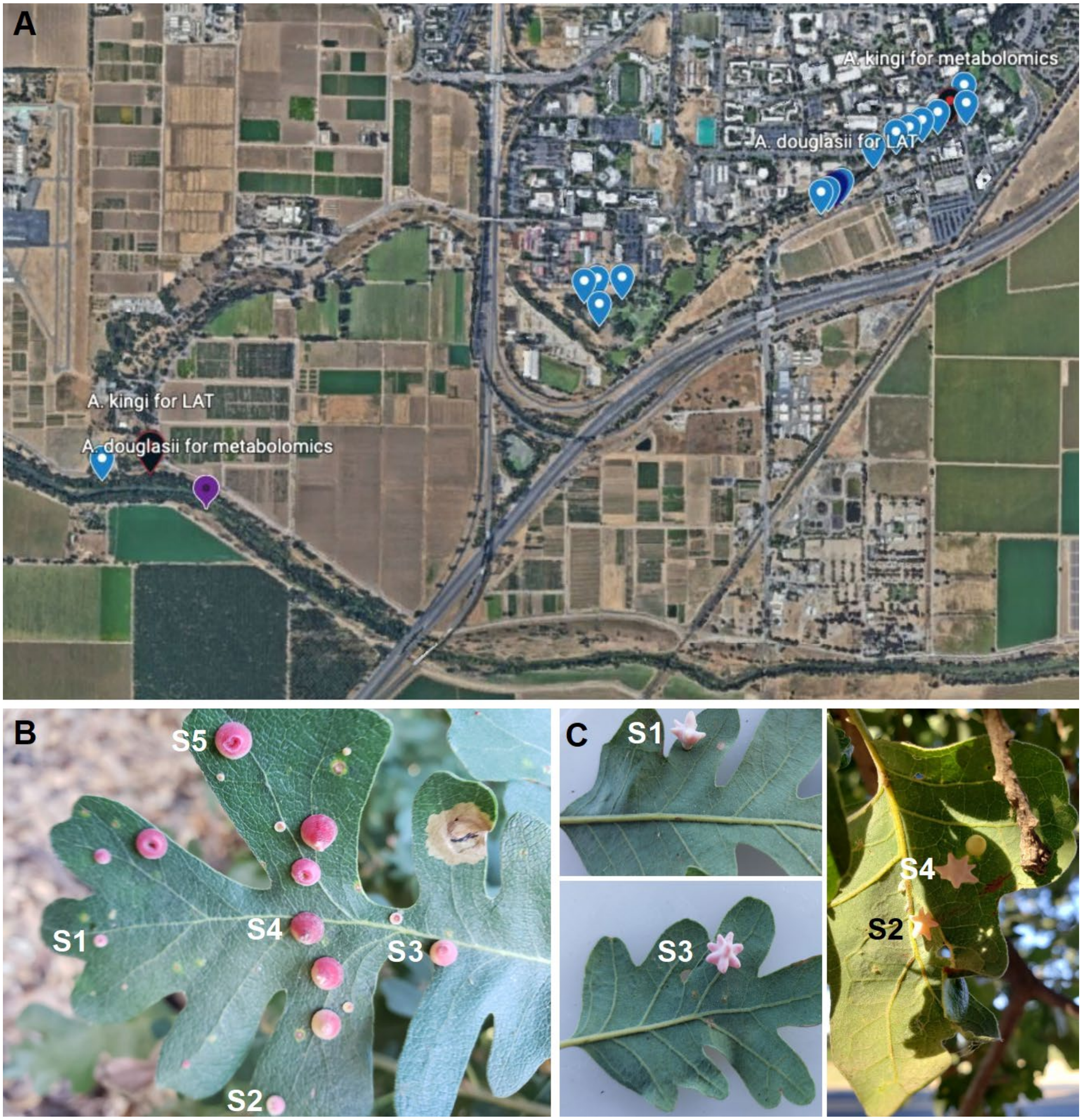


**Supplemental Figure S1: Gall map and photographs**. A: Map of collection sites. Round tags indicate individual oak trees from which galls were harvested, red tags indicate harvests of galls induced by *Andricus kingi* that were used in further experiments, purple tags indicate harvests of galls induced by *Antrol douglasii* in further experiments. blue tags indicate other harvests which may have been used for microscopy or other analyses. For each experiment, all galls of a particular type were harvested from one tree on one day. B: photograph of cone galls in the field, demonstrating their high density on the leaf. Labels to the left of selected galls indicate their growth stage. C: photographs of urchin galls in the field. Labels to the left of galls indicate their growth stage. The example of S3 here is the same image used in Figure 1B.

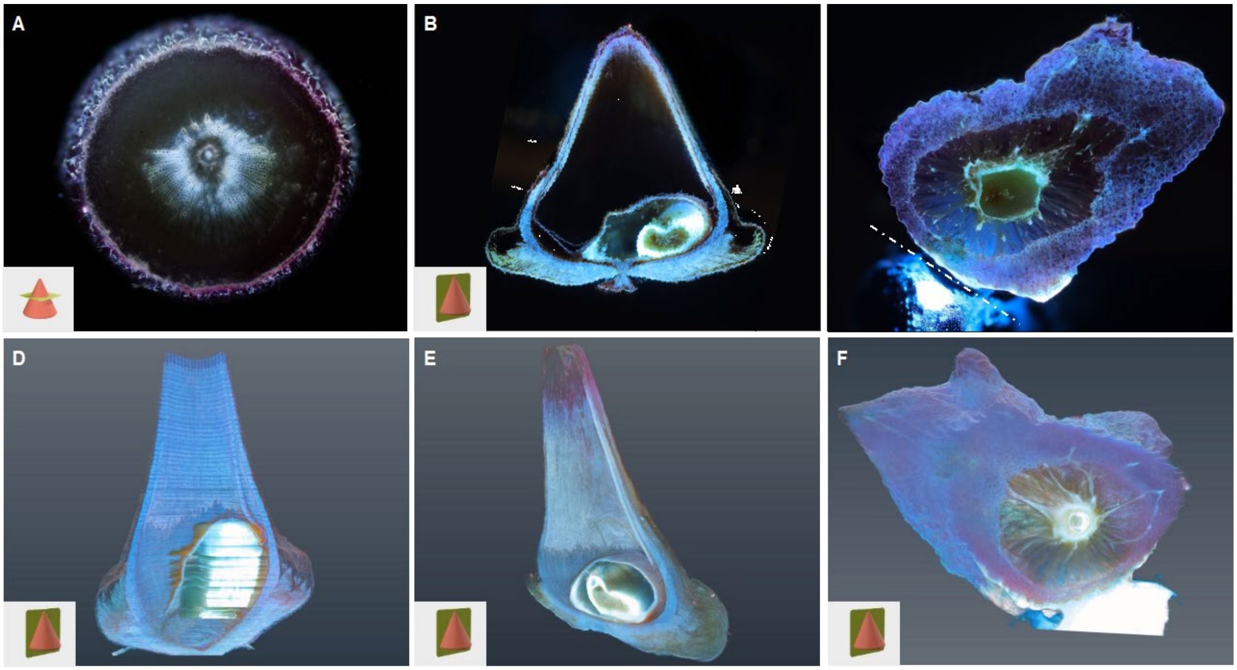


**Supplemental Figure S2: Laser ablation tomography sections and three-dimensional models.** A: Transverse section of cone gall. B: Longitudinal sectional of cone gall. C: longitudinal section of urchin gall. The object below and left of the dashed white line is the sample support rod, not part of the gall. D: three-dimensional model of cone gall imaged in transverse sections (apparent horizontal lines are an artifact of aliasing in assembling individual slices and stochastic variance in image brightness), corresponds to supplemental movie 1. E: three-dimensional model of cone gall imaged in longitudinal sections, corresponds to supplemental movie 2. F: three-dimensional model of urchin gall, corresponds to supplemental movie 3. Inset diagrams in lower left corner indicate orientation as viewed, which corresponds to view as imaged with the exception of D, where view shown here is orthogonal to the imaging plane.

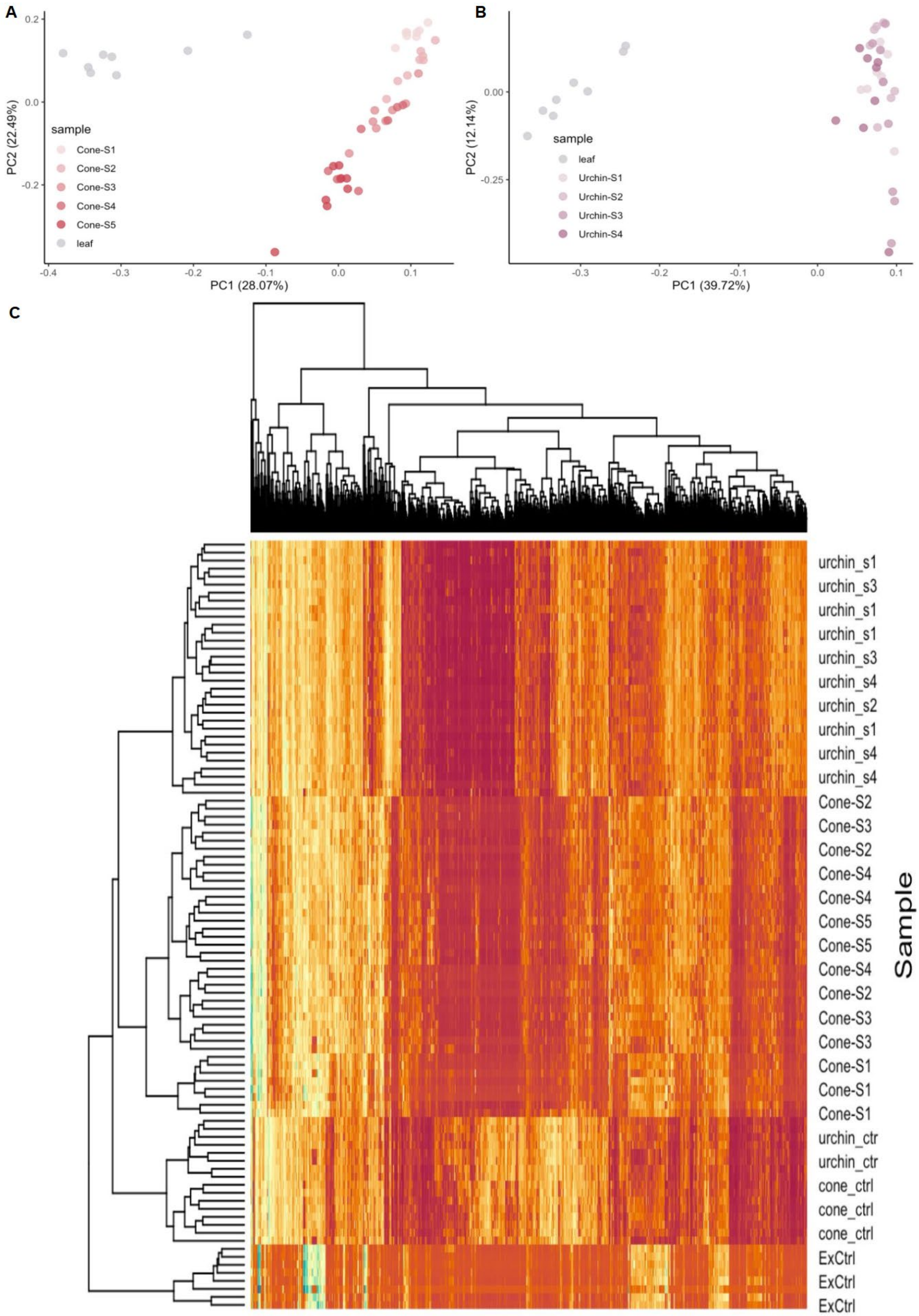
A
B
C
PC1 (28.07%)
PC2 (22.49%)
sample
Cone-S1
Cone-S2
Cone-S3
Cone-S4
Cone-S5
leaf
PC1 (39.72%)
PC2 (12.14%)
Urchin-S1
Urchin-S2
Urchin-S3
Urchin-S4
urchin_s1
urchin_s3
urchin_s1
urchin_s1
urchin_s3
urchin_s4
urchin_s2
urchin_s1
urchin_s4
urchin_s4
Cone-S2
Cone-S3
Cone-S2
Cone-S4
Cone-S4
Cone-S5
Cone-S5
Cone-S4
Cone-S2
Cone-S3
Cone-S3
Cone-S1
Cone-S1
Cone-S1
urchin_ctr
urchin_ctr
cone_ctrl
cone_ctrl
cone_ctrl
ExCtrl
ExCtrl
ExCtrl
Sample

**Supplemental Figure S3: Gall metabolomes cluster by developmental stage**. A: PCA of the developmental stages of cone galls. B: PCA of the developmental stages of urchin galls. C: Heatmap of untargeted metabolomics with dendrogram. Rows are samples, columns are metabolites.

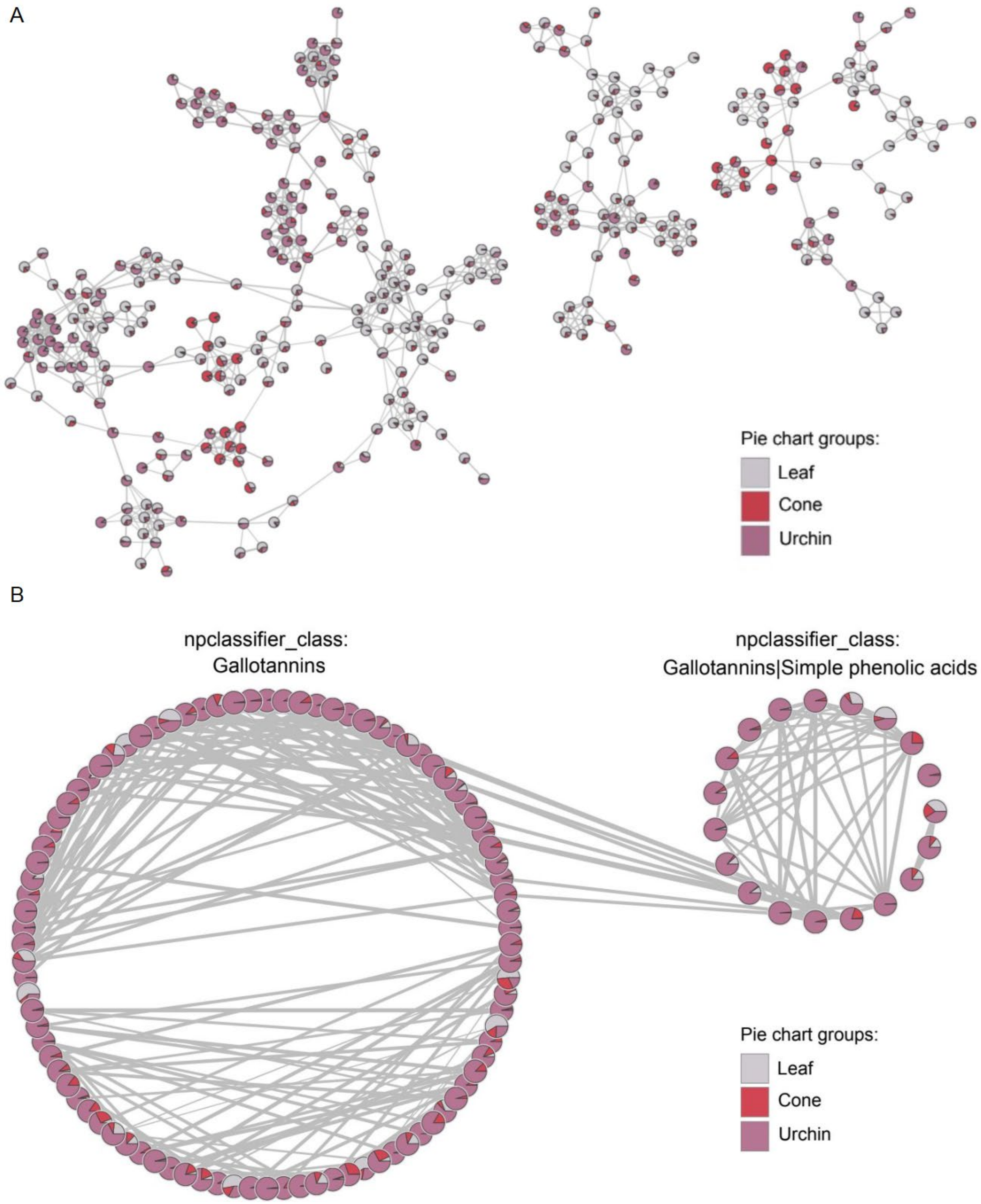


**Supplemental Figure S4: Gall metabolomes are distinct.** A: Network from untargeted metabolomics. Interactive networks with putative identifications of mass features available at NDExBio. B: Network of mass features classified as gallotannins by NPClassifier.

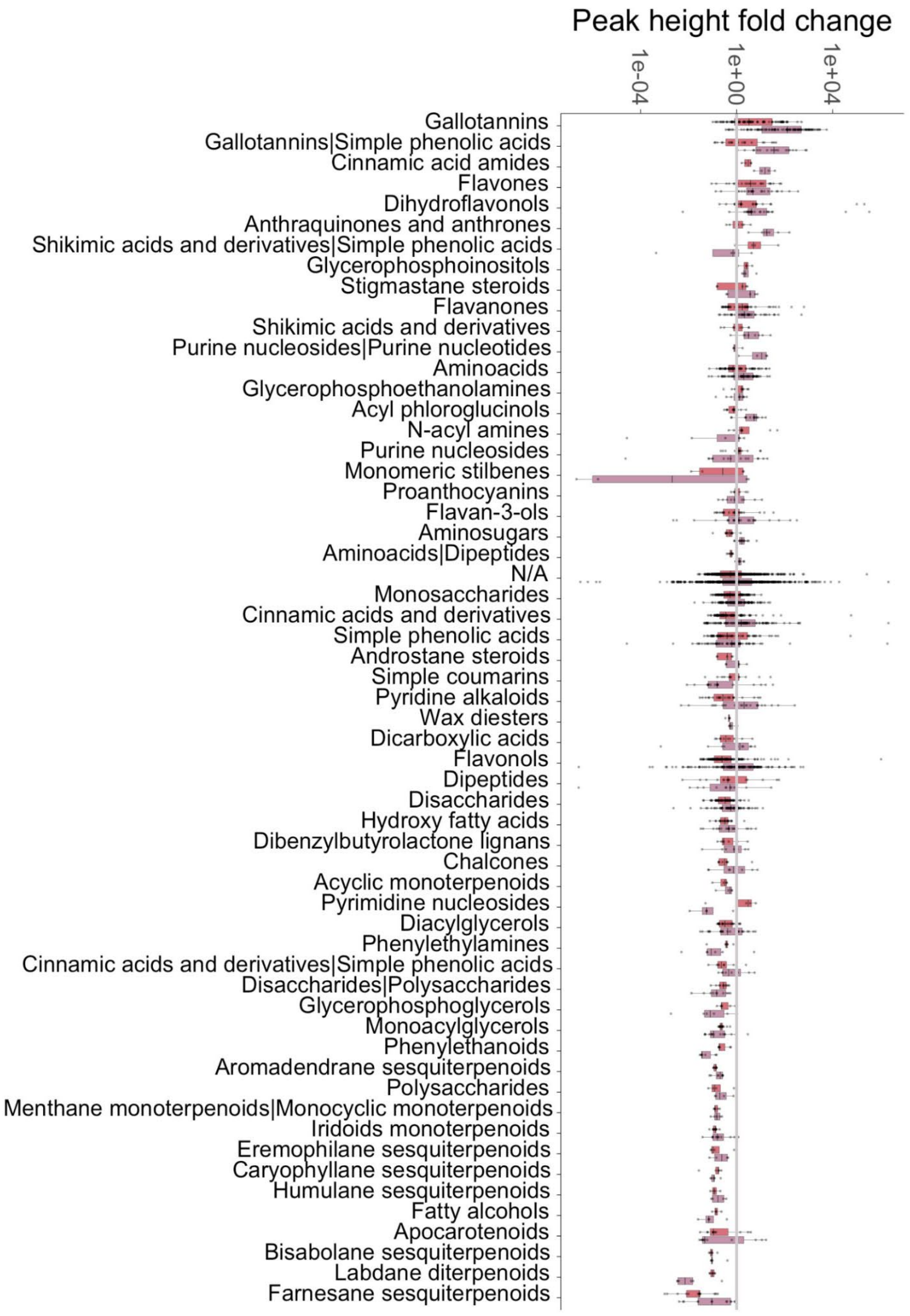


**Supplemental Figure S5**: **Galls differ in chemical class concentration** Peak height fold change for all NPClassifier categories for both types of gall compared to leaf tissue. Boxplot elements: center line, median; box limits, upper and lower quartiles; whiskers, 1.5x interquartile range; points, outliers. X axis is log10 transformed.

A

| | EMA Compound Info |
|---|---|
| Name: | ABSCISIC ACID |
| Label: | |
| Formula: | C15H20O4 |
| Polarity: | positive |
| Monoisotopic Mass: | 264.13615912 |
| Theoretical M/Z: | 247.1328591 |
| Adduct: | [M+H-H2O]+ |

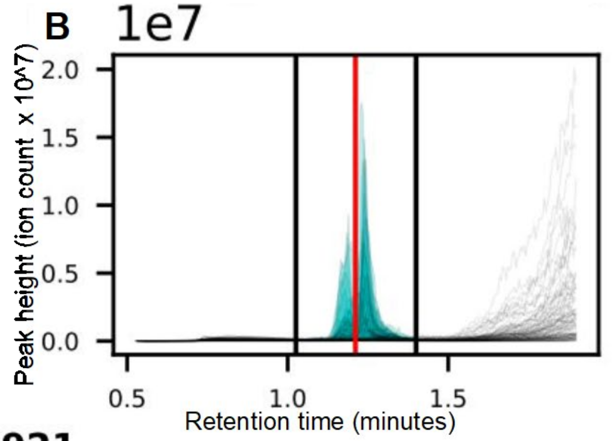


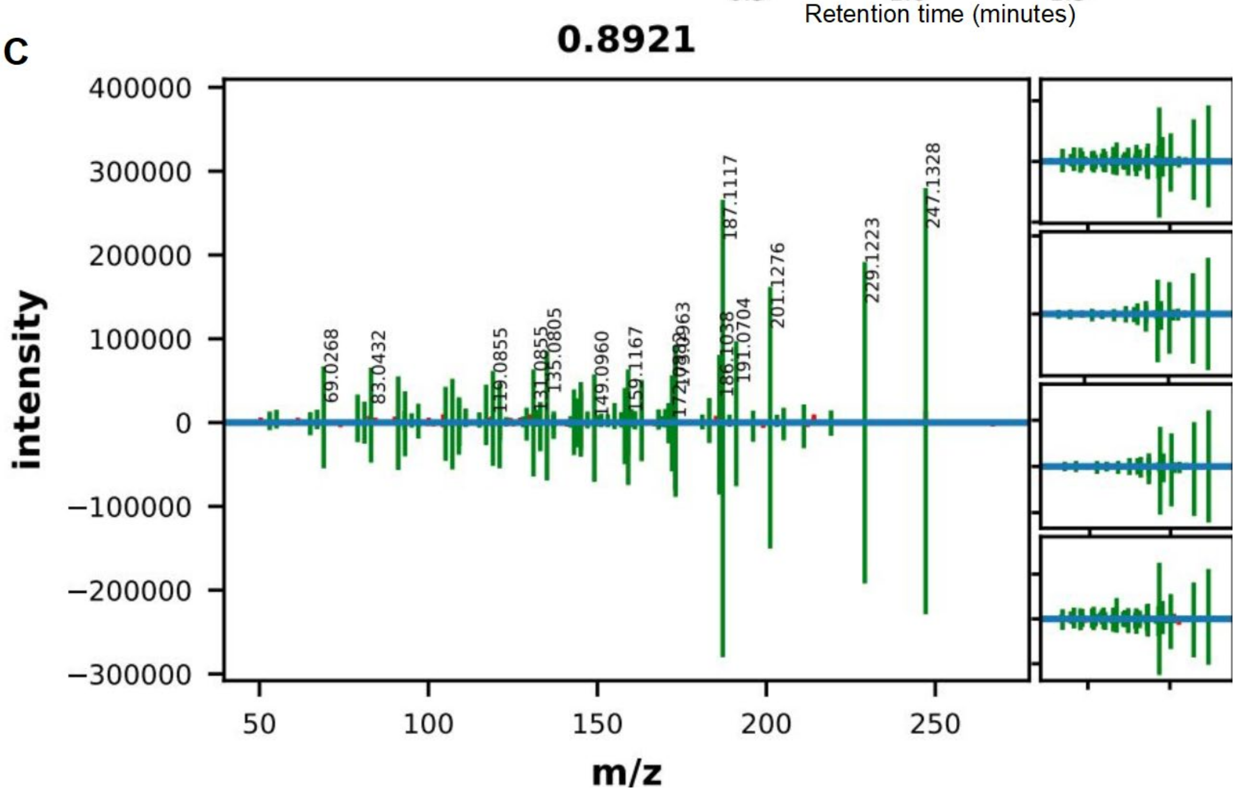


**Supplemental Figure S6: MS-MS details for abscisic acid.** A: Information table. B: Extracted ion chromatogram across all files. The predicted retention time is the red vertical line, the black vertical lines are the integration bounds. C: MS-MS mirror plot, our data on top and standard MS-MS fragmentation pattern on bottom. Cosine score = 0.8921. Panels to the right are the next four highest scoring comparisons.

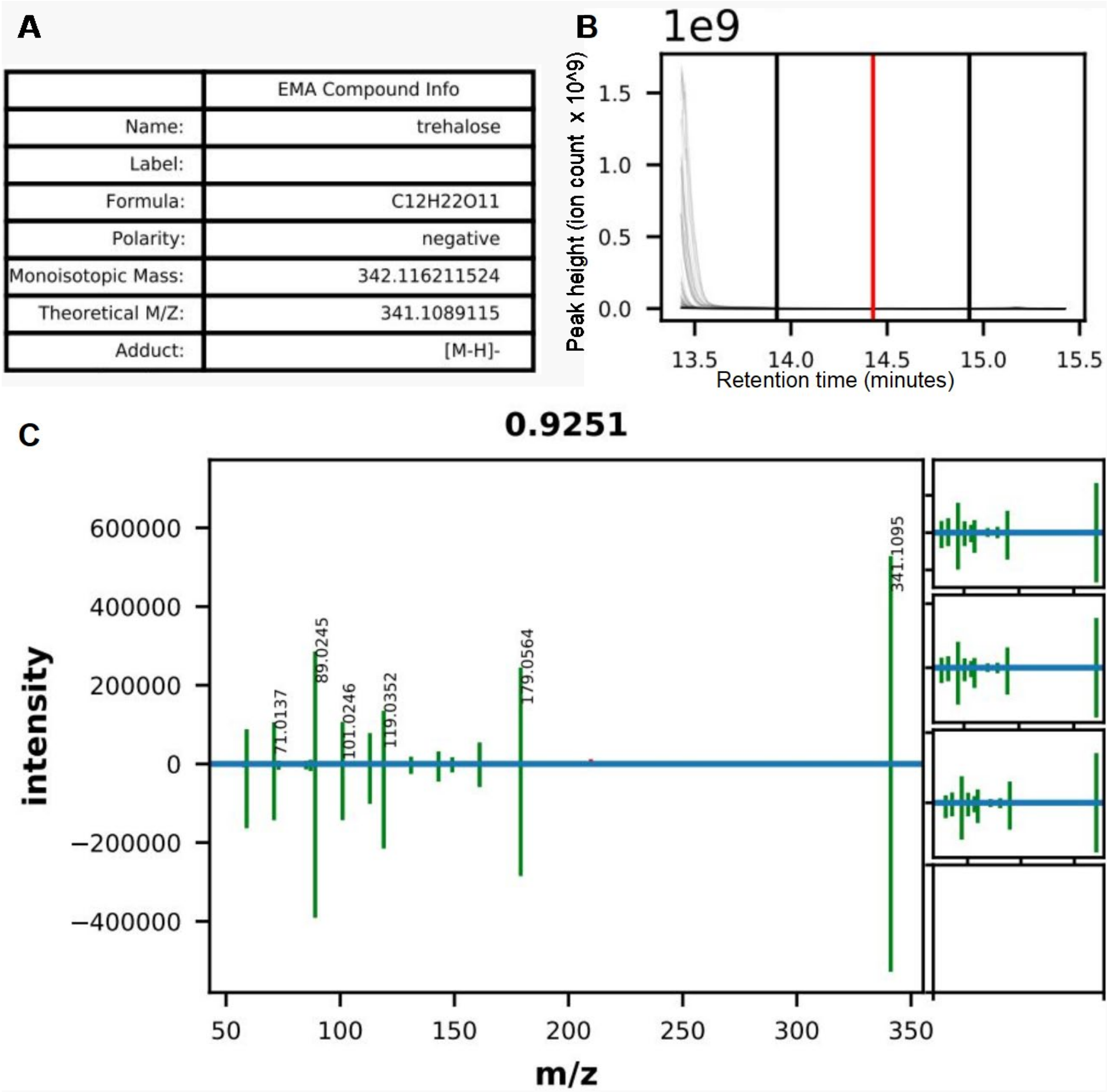


**Supplemental Figure S7: MS-MS details for trehalose.** A: Information table. B: Extracted ion chromatogram across all files. The predicted retention time is the red vertical line, the black vertical lines are the integration bounds. Due to software constraints, this graph is too zoomed out on the Y axis because of another peak around 13.2 minutes. The maximum peak intensity for trehalose is 7.65 x 10^6, which is not visible due to the Y axis scaling. The peak is at 14.42 minutes (very close to the predicted retention time), and the integration bounds are set well, as can be seen in Dataset S5. C: MS-MS mirror plot, our data on top and standard MS-MS fragmentation pattern on bottom. Cosine score = 0.9251. Panels to the right are the next four highest scoring comparisons. In this case, only alternative three matches were found by the software.

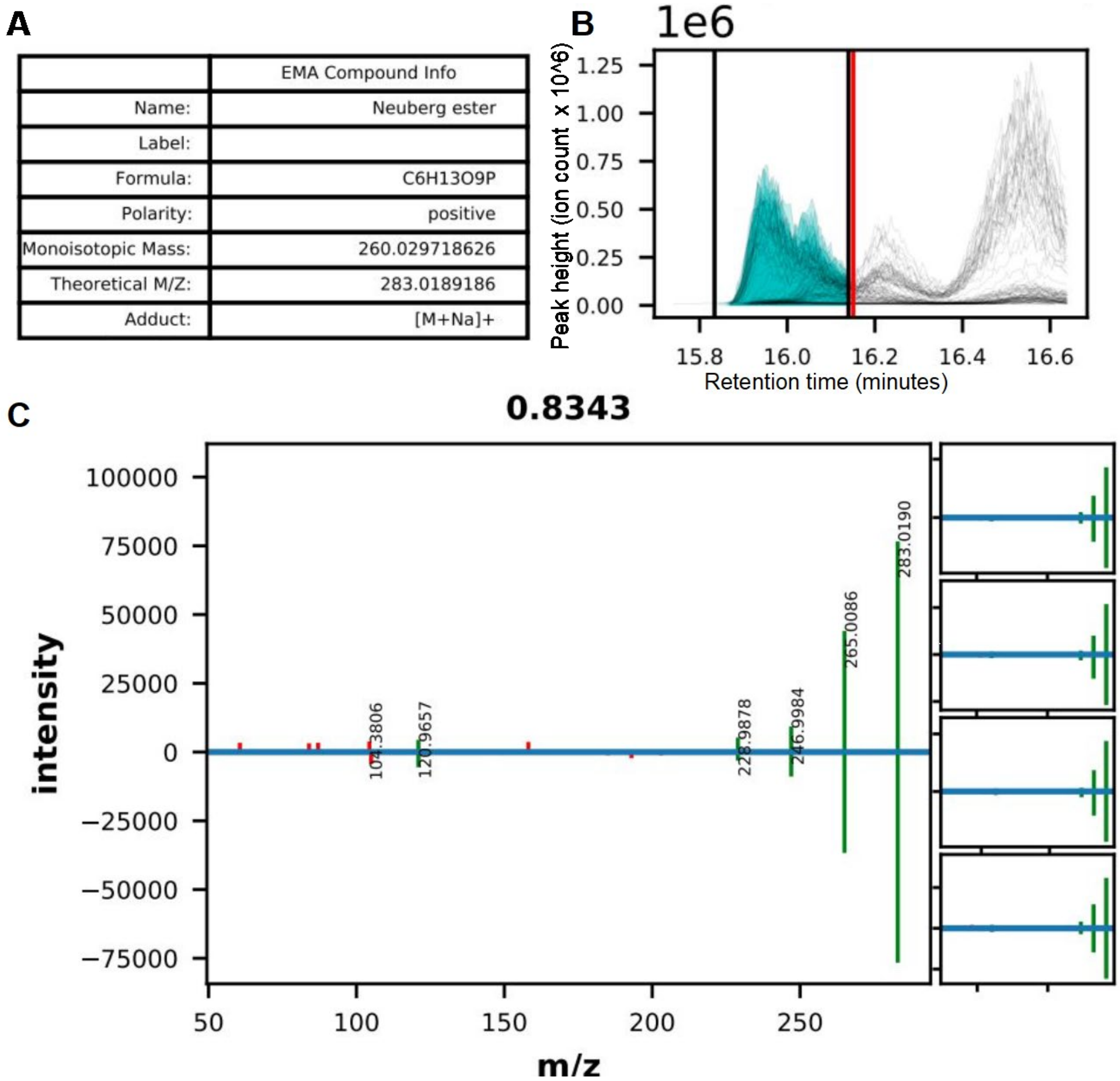


**Supplemental Figure S8: MS-MS details for hexose phosphate.** A: Information table. While the software identified this feature as Neuberg ester meaning Fructose-6-phosphate, we are not confident of this specific isomeric identification, only that this is a hexose phosphate. B: Extracted ion chromatogram across all files. The predicted retention time is the red vertical line, the black vertical lines are the integration bounds. C: MS-MS mirror plot, our data on top and standard MS-MS fragmentation pattern on bottom. Cosine score = 0.8343. Panels to the right are the next four highest scoring comparisons.

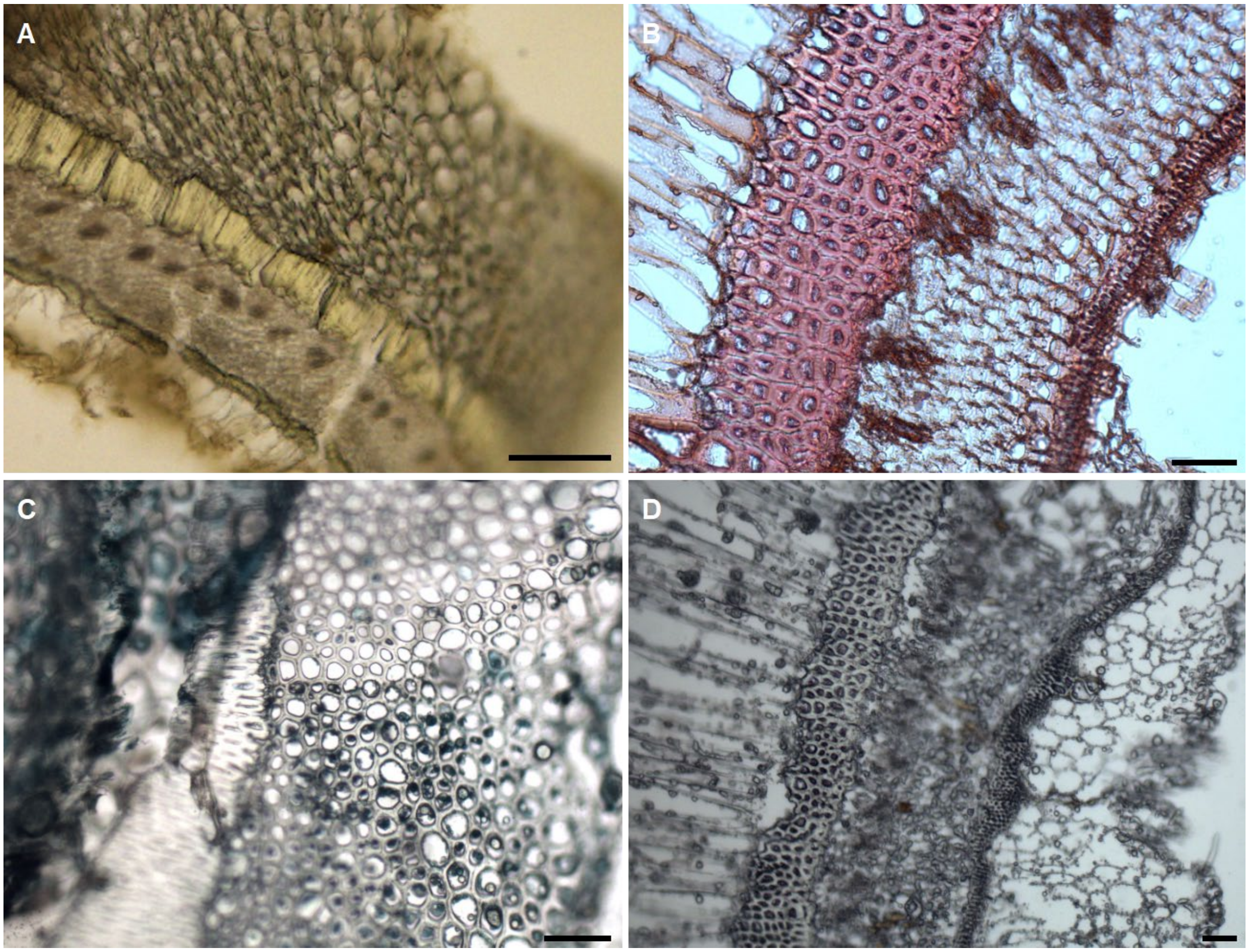


**Supplemental Figure S9: Sample of additional histological stains.** A: Cone gall stained with Mäule stain. Scale bar = 100 µm. B: Cone gall stained with Safranin O. Scale bar = 50 µm C: Cone gall stained with FastGreen. Scale bar = 50 µm D: Unstained cone gall. Scale bar = 50 µm

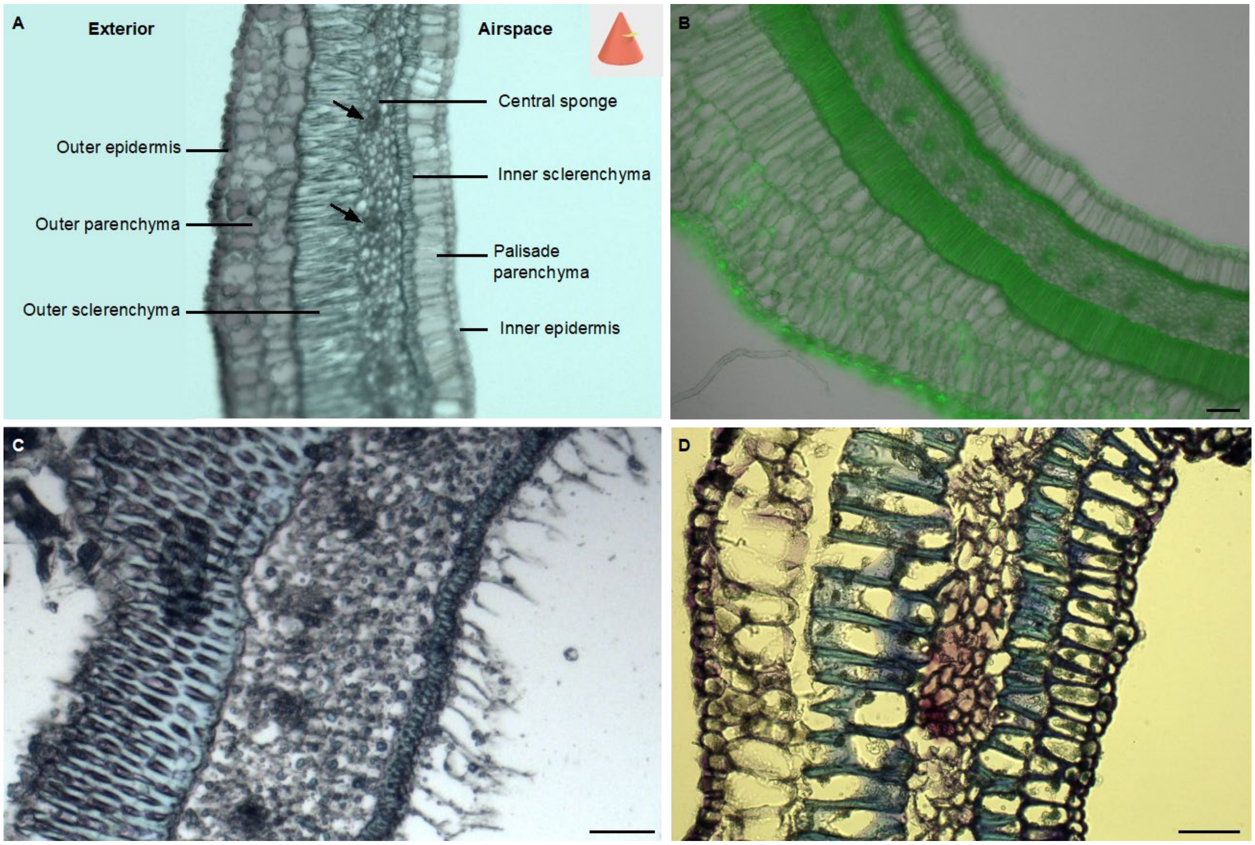


**Supplemental Figure S10: Micrographs of cone galls and map of cell layers** A: Transverse section of cone gall with cell layers labeled. There are 7 distinct cell layers, which can be seen by their morphology and differential uptake of stains. Arrows indicate bundles which are moderately lignified. Outer sclerenchyma and inner sclerenchyma are heavily lignified. B: Transverse section of cone gall imaged by autofluorescence using GFP filter. Scale bar = 50 μm C: 12 μm cryosection stained with toluidine blue O. The thick cell walls of the outer sclerenchyma are stained blue, and the bundles are also clearly visible as dark patches in the central sponge layer. Scale bar = 50 μm. D: 12 μm cryosection stained longer with toluidine blue O. The outer epidermis and parenchyma are stained purple, outer and inner sclerenchyma stained blue, and palisade parenchyma and inner epidermis are stained a dark violet. Scale bar = 50 μm

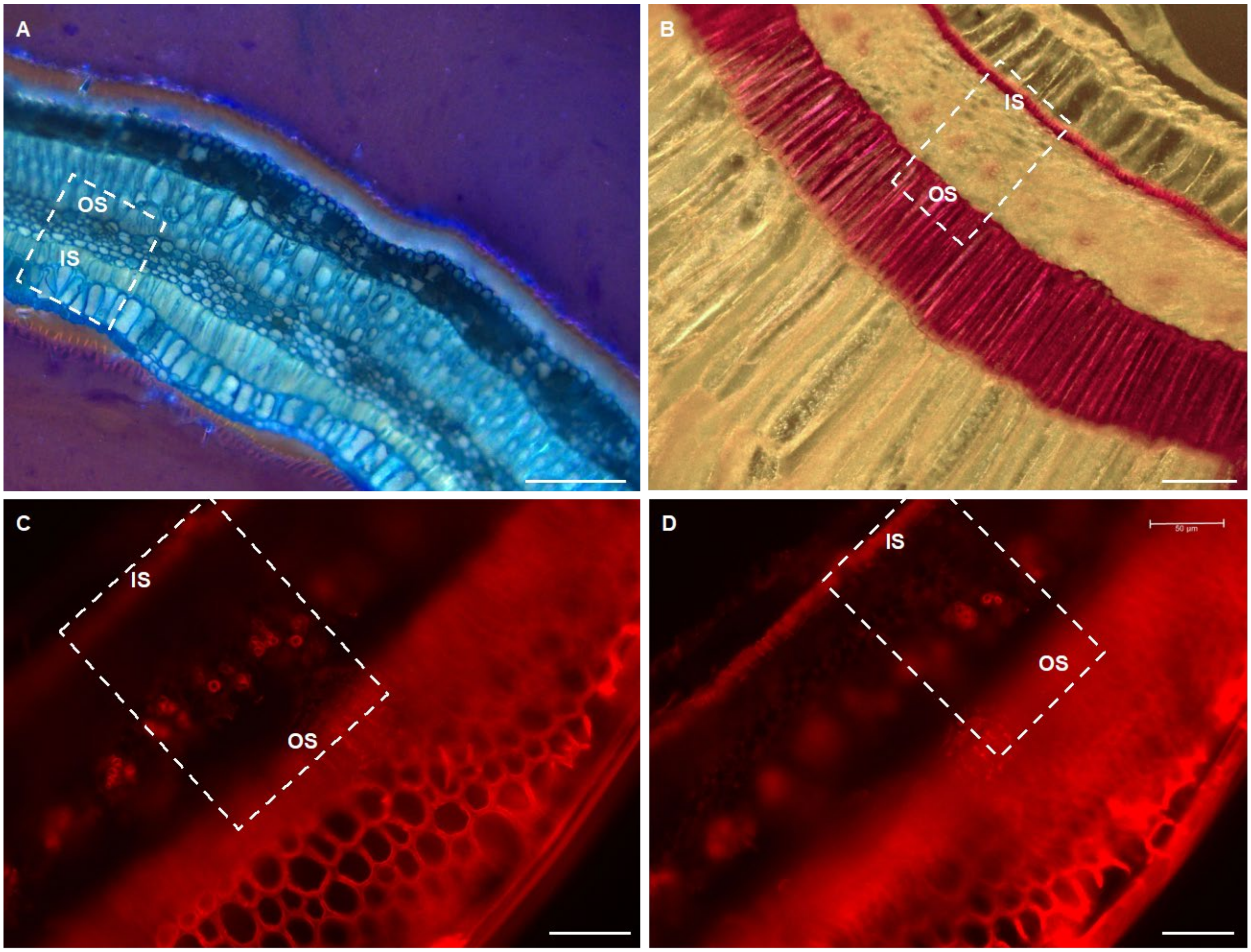


**Supplemental Figure S11: Transverse sections of red cone galls.** A: Cone gall stained with toluidine blue O. Scale bar = 100 µm (half-sized bar used to indicate 50 µm in Fig 4C). B: Cone gall stained with Wiesner stain, same image as used in Figure 3A. Scale bar = 100 µm (half-sized bar used to indicate 50 µm in Fig 4C). C: Transverse section of cone gall immunostained with LM10. D: Transverse section of cone gall immunostained with LM10. Dashed rectangle goes beyond the edge of the micrograph, explaining the dark gray triangle in bottom right of the rightmost image in Fig 4C. Scale bar = 50 µm. For all micrographs, the dotted rectangle shows the portion of the image used in Fig 4C. OS = outer sclerenchyma, IS = inner sclerenchyma.
